# Association between smoking cessation and non-alcoholic fatty liver disease using NAFLD liver fat score

**DOI:** 10.3389/fpubh.2023.1015919

**Published:** 2023-02-17

**Authors:** Yun Seo Jang, Hye Jin Joo, Yu Shin Park, Eun-Cheol Park, Sung-In Jang

**Affiliations:** ^1^Department of Public Health, Graduate School, Yonsei University, Seoul, Republic of Korea; ^2^Institute of Health Services Research, Yonsei University, Seoul, Republic of Korea; ^3^Department of Preventive Medicine, Yonsei University College of Medicine, Seoul, Republic of Korea

**Keywords:** smoking, smoking behavior, smoking history, smoking cessation, tobacco, pack-years, nonalcoholic fatty liver disease

## Abstract

**Background:**

Smoking is well known to be associated with a higher prevalence and incidence of liver diseases such as advanced fibrosis. However, the impact of smoking on developing nonalcoholic fatty liver disease remains controversial, and clinical data on this is limited. Therefore, this study aimed to investigate the association between smoking history and nonalcoholic fatty liver disease (NAFLD).

**Methods:**

Data from the Korea National Health and Nutrition Examination Survey 2019-2020 were used for the analysis. NAFLD was diagnosed according to an NAFLD liver fat score of >-0.640. Smoking status was classified as into nonsmokers, ex-smokers, and current smokers. Multiple logistic regression analysis was conducted to examine the association between smoking history and NAFLD in the South Korean population.

**Results:**

In total, 9,603 participants were enrolled in this study. The odds ratio (OR) for having NAFLD in ex-smokers and current smokers in males was 1.12 (95% confidence interval [CI]: 0.90–1.41) and 1.38 (95% CI: 1.08–1.76) compared to that in nonsmokers, respectively. The OR increased in magnitude with smoking status. Ex-smokers who ceased smoking for <10 years (OR: 1.33, 95% CI: 1.00–1.77) were more likely to have a strong correlation with NAFLD. Furthermore, NAFLD had a dose-dependent positive effect on pack-years, which was 10 to 20 (OR: 1.39, 95% CI: 1.04–1.86) and over 20 (OR: 1.51, 95% CI: 1.14–2.00).

**Conclusion:**

This study found that smoking may contribute to NAFLD. Our study suggests cessation of smoking may help management of NAFLD.

## Introduction

Nonalcoholic fatty liver disease (NAFLD) is the most common chronic liver disease. It is a condition in which neutral fat accumulates excessively in the liver (1, 2). Although there are some differences in its frequency from country to country, it has been reported that 6.3 to 33% and an average of approximately 20% of patients worldwide have been affected by the disease (3). The prevalence of NAFLD is rapidly increasing in Asian countries due to the increase in Westernized eating habits, obesity, and the diabetic population (4, 5). In addition, between 10 and 29% of patients with nonalcoholic fatty hepatitis develop cirrhosis within 10 years and between 4 and 27% of patients develop liver cancer (6, 7). Furthermore, patients with NAFLD have a higher mortality rate than healthy controls, and the mortality rate related to liver disease is also high (8–11). Therefore, NAFLD must be managed immediately due to its expected serious public health burden and significant social costs (12, 13).

Tobacco smoke contains more than 7,000 chemicals, of which at least 250 are known to be harmful, such as ammonia and hydrogen cyanide (14, 15). Smoking is closely related to chronic diseases, such as cardiovascular diseases, cancer, and type 2 diabetes (16–19), which are also related to NAFLD (20–22). Previous studies have suggested smoking is associated with increased prevalence and incidence of liver diseases (23, 24). In particular, it has been reported to be an independent risk factor for the progression of advanced fibrosis in patients with primary biliary cirrhosis (23) and chronic hepatitis C (24).

A positive association between smoking and NAFLD has been continuously reported (25–27). An experimental study suggested cigarettes accelerated the progression of NAFLD in obese mice-fed diets (25). Furthermore, a study conducted in mice without apolipoprotein E, a condition wherein fatty liver is easily occurs, found that nicotine in electronic cigarettes (e-cigarettes) causes genetic mutations and promotes NAFLD outbreaks (26). Other studies have shown that the activation of sterol regulatory element-binding proteins (SREBPs), which stimulate the synthesis of fatty acids in the liver, is associated with NAFLD (27). These studies provided evidence of the mechanism of the relationship between smoking and the prevalence of NAFLD. However, most studies are experimental studies conducted on animals, and there are not many studies conducted on humans.

Therefore, this study aimed to examine the association between smoking history and NAFLD in a representative population and to explain whether smoking behavior plays a potential role in developing NAFLD.

## Materials and methods

### Data

The study used cross-sectional data from the 2019–2020 National Health and Nutrition Examination Survey (KNHANES), conducted by the Korea Centers for Disease Control and Prevention Agency (KDCA). The KNAHENS is a self-report survey using a stratified, multistage, cluster sampling design conducted annually for South Koreans of all ages to evaluate the health and nutritional status. The survey provides data for the evaluation and development of health policies and programs and does not require ethical approval from the ethics review board, as the KNHANES conforms to the Declaration of Helsinki.

### Study population

**Figure 1:**
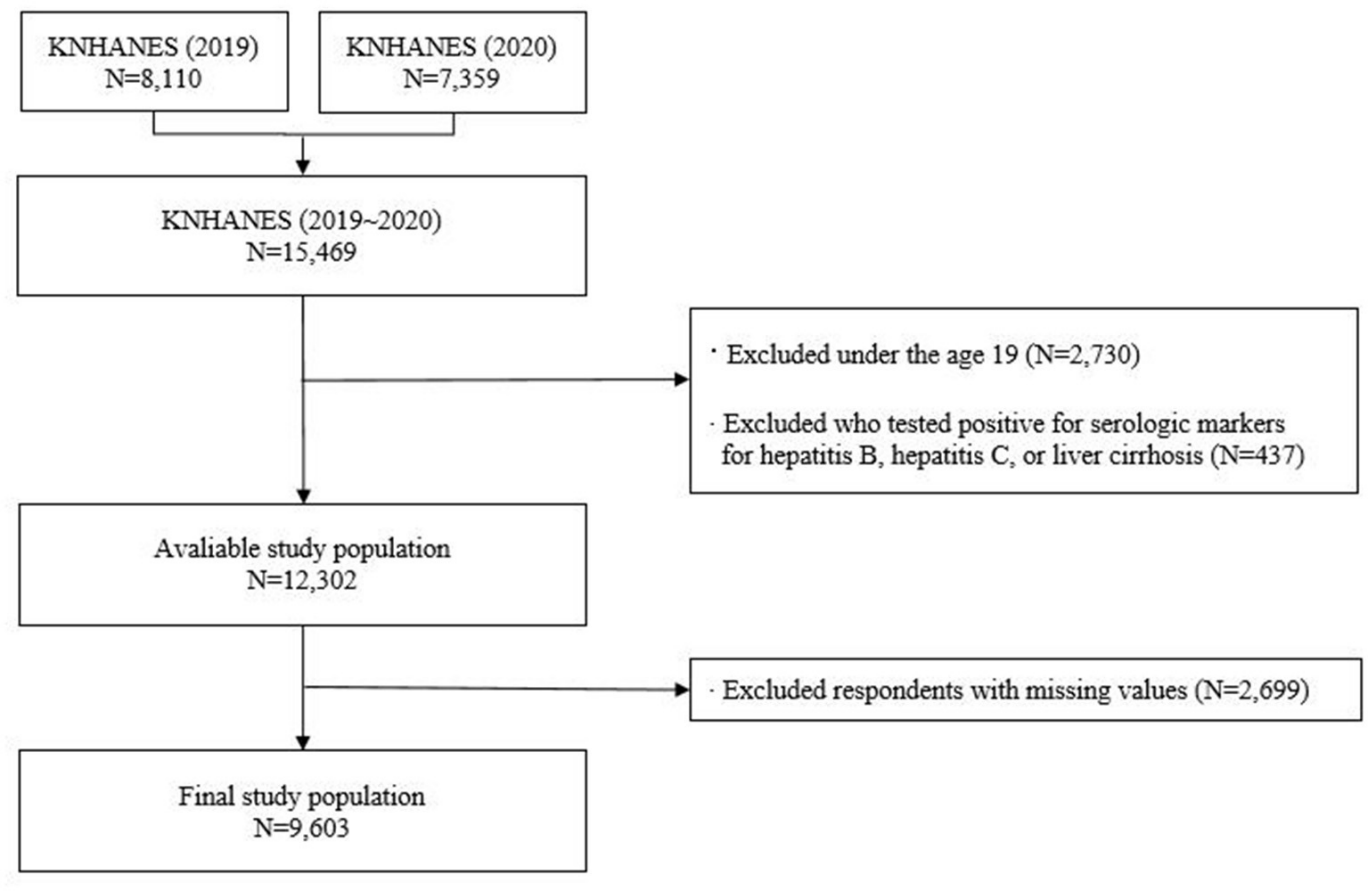
Flowchart of the study participants showing the inclusion and exclusion.

Of the 15,469 survey participants, we excluded those under 19 years of age and those who did not participate in a KNHANES smoking questionnaire survey (*n* = 2,730). Furthermore, participants who tested positive for serologic markers for liver diseases (hepatitis B, hepatitis C, and liver cirrhosis) were excluded (*n* = 437). Participants with missing data were also excluded (*n* = 2,699). Consequently, a final sample of 9,603 participants was analyzed in this study (Figure 1). As a study that examined the effects of smoking on NAFLD, participants with alcohol-related fatty liver disease were also excluded based on their biochemical and clinical profiles.

### Variables

The main dependent variable was the prevalence of NAFLD. NAFLD was diagnosed according to the NAFLD liver fat score developed by the Department of Medicine and the Minerva Medical Research Institute at Helsinki University (28). The NAFLD liver fat score formula was derived using a multivariate logistic regression model using metabolic syndrome, type 2 diabetes, fasting insulin (fS), serum aspartate aminotransferase (AST) ratio, and AST to serum alanine aminotransferase (ALT) ratio (28): NAFLD liver fat score = −0.89 + 1.18 × metabolic syndrome (yes = 1 / no = 0) + 0.45 × diabetes (yes = 2 / no = 0) + 0.15 × fS-insulin (mu/L) + 0.04 × fS-AST (U/L) – 0.94 × AST/ALT. Participants were considered to have NAFLD if their liver fat score of NAFLD was >−0.640 as the optimal cutoff point (28).

The primary independent variable was the smoking status of the participants, which was divided into three groups: (1) nonsmokers, (2) ex-smokers, and (3) current smokers. This was defined based on the questions: 'Do you currently smoke conventional cigarettes?'; “Do you currently smoke e-cigarettes?”. This classification was the same as that of a previous study that used the same research tool to investigate smoking behavior (29).

The covariates included demographic factors (sex, age, marital status, and educational level), socioeconomic factors (household income, region, and occupational categories), behavioral health patterns (current drinking status, physical activity), and health-related factors (body mass index (BMI), diagnosis of hypertension, and diagnosis of diabetes).

### Statistical analysis

All estimates were calculated using sample weight procedures to improve representativeness and generalize the data. Clusters and strata were assigned to the study population. The general characteristics of the study group, represented by frequencies and percentages for categorical variables, means and standard deviations for continuous variables, were based on descriptive analysis. After adjusting for covariates, a multiple logistic regression analysis was performed to assess the relationship between smoking and NAFLD. Subgroup analyzes were also performed according to age, current drinking status, physical activity, BMI, and diagnosis of hypertension and diabetes. Furthermore, we also performed a subgroup analysis for a more complete analysis of smoking behavior, including smoking cessation status (SCS) and pack years. All statistical analyses were performed using SAS version 9.4 (SAS Institute Inc., Cary, NC, USA).

## Results

Table 1 shows the characteristics of the study population. Of the 9,603 participants, 4,063 were men (42.3%) and 5,540 were women (57.7%). Among males, 1,249 (30.7%) were current smokers, 1,674 (41.2%) were ex-smokers, and 1,140 (28.1%) were nonsmokers. Among the females, 259 (4.7%) were current smokers, 312 (5.6%) were ex-smokers, and 4,969 (89.7%) were nonsmokers. In total, 1,433 (35.3%) men and 1,278 (23.1%) women reported NAFLD.

**Table 1:** General characteristics of the study population.

**Variables**		**Nonalcoholic fatty liver disease (NAFLD)**													
		**Male**							**Female**						
		**Total**		**Yes**		**No**		**P-** ***value***	**Total**		**Yes**		**No**		**P-** ***value***
		**N**	**%**	**N**	**%**	**N**	**%**		**N**	**%**	**N**	**%**	**N**	**%**	
**Total (** ***N*** **=9,603)**		4,063	100.0	1,433	35.3	2,630	64.7		5,540	100.0	1,278	23.1	4,262	76.9	
**Smoking Behavior**								0.0014							0.5628
	Nonsmoker	1,140	28.1	354	31.1	786	68.9		4,969	89.7	1,156	23.3	3,813	76.7	
	Ex-smoker	1,674	41.2	629	37.6	1,045	62.4		312	5.6	65	20.8	247	79.2	
	Current smoker	1,249	30.7	450	36.0	799	64.0		259	4.7	57	22.0	202	78.0	
**Age (Mean, SD)**		51.6	17.2	52.2	15.9	51.3	17.9	< 0.0001	51.8	16.4	49.8	16.3	58.6	14.9	< 0.0001
**Marital status**							0.0185							0.1441
	Married	2,884	71.0	1,043	36.2	1,841	63.8		3,664	66.1	835	22.8	2,829	77.2	
	Divorced, Separated	166	4.1	67	40.4	99	59.6		343	6.2	94	27.4	249	72.6	
	Single, widow	1,013	24.9	323	31.9	690	68.1		1,533	27.7	349	22.8	1,184	77.2	
**Educational level**								0.8249							< 0.0001
	Middle school or below	878	21.6	317	36.1	561	63.9		1,715	31.0	621	36.2	1,094	63.8	
	High school	1,472	36.2	513	34.9	959	65.1		1,787	32.3	381	21.3	1,406	78.7	
	College or over	1,713	42.2	603	35.2	1,110	64.8		2,038	36.8	276	13.5	1,762	86.5	
**Household income**								0.6334							< 0.0001
	Low	651	16.0	224	34.4	427	65.6		1,046	18.9	353	33.7	693	66.3	
	Mid-low	985	24.2	364	37.0	621	63.0		1,360	24.5	334	24.6	1,026	75.4	
	Mid-high	1,129	27.8	396	35.1	733	64.9		1,488	26.9	297	20.0	1,191	80.0	
	High	1,298	31.9	449	34.6	849	65.4		1,646	29.7	294	17.9	1,352	82.1	
**Region**								0.2872							< 0.0001
	Metropolitan	1,720	42.3	584	34.0	1,136	66.0		2,466	44.5	502	20.4	1,964	79.6	
	Urban	1,505	37.0	540	35.9	965	64.1		2,034	36.7	459	22.6	1,575	77.4	
	Rural	838	20.6	309	36.9	529	63.1		1,040	18.8	317	30.5	723	69.5	
**Occupational categories**								0.6403							< 0.0001
	White	1,155	28.4	422	36.5	733	63.5		1,263	22.8	174	13.8	1,089	86.2	
	Pink	404	9.9	137	33.9	267	66.1		835	15.1	191	22.9	644	77.1	
	Blue	1,308	32.2	449	34.3	859	65.7		822	14.8	217	26.4	605	73.6	
	Inoccupation	1,196	29.4	425	35.5	771	64.5		2,620	47.3	696	26.6	1,924	73.4	
**Current drinking status**								0.0315							< 0.0001
	Never or occasionally	1,273	31.3	435	34.2	838	65.8		3,310	59.7	892	26.9	2,418	73.1	
	2–4 times/month	1,478	36.4	498	33.7	980	66.3		1,633	29.5	297	18.2	1,336	81.8	
	2–4 times/week	1,312	32.3	500	38.1	812	61.9		597	10.8	89	14.9	508	85.1	
**Physical activity**								< 0.0001							< 0.0001
	Adequate	1,906	46.9	613	32.2	1,293	67.8		2,216	40.0	425	19.2	1,791	80.8	
	Inadequate	2,157	53.1	820	38.0	1,337	62.0		3,324	60.0	853	25.7	2,471	74.3	
**BMI**								< 0.0001							< 0.0001
	Normal	1,155	28.4	132	11.4	1,023	88.6		2,494	45.0	175	7.0	2,319	93.0	
	Underweight	92	2.3	3	3.3	89	96.7		272	4.9	6	2.2	266	97.8	
	Overweight	1,069	26.3	274	25.6	795	74.4		1,116	20.1	268	24.0	848	76.0	
	Obesity of stage 1	1,472	36.2	796	54.1	676	45.9		1,351	24.4	597	44.2	754	55.8	
	Obesity of stages 2&3	275	6.8	228	82.9	47	17.1		307	5.5	232	75.6	75	24.4	
**Diagnosis of hypertension**								< 0.0001							< 0.0001
	Yes	1,105	27.2	530	48.0	575	52.0		1,290	23.3	577	44.7	713	55.3	
	No	2,958	72.8	903	30.5	2,055	69.5		4,250	76.7	701	16.5	3,549	83.5	
**Diagnosis of diabetes**								< 0.0001							< 0.0001
	Yes	466	11.5	299	64.2	167	35.8		500	9.0	339	67.8	161	32.2	
	No	3,597	88.5	1,134	31.5	2,463	68.5		5,040	91.0	939	18.6	4,101	81.4	
**Year**								0.0018							0.1354
	2019	2,088	51.4	689	33.0	1,399	67.0		2,932	52.9	653	22.3	1,983	77.7	
	2020	1,975	48.6	744	37.7	1,231	62.3		2,608	47.1	625	24.0	2,279	76.0	

Table 2 presents the results of the multiple regression analysis for the relationship between smoking and NAFLD stratified by sex after adjusting for all covariates. Among male participants, the odds ratios (OR) for NAFLD among ex-smokers and current smokers were 1.12 (95% confidence interval [CI]: 0.90–1.41) and 1.38 (95% CI: 1.08–1.76), respectively. In women, the OR for NAFLD among ex-smokers and current smokers were 1.32 (95% CI: 0.86–2.01) and 1.18 (95% CI: 0.76–1.83), respectively. Ex-smokers and current smokers exhibited an increasing trend of OR for NAFLD compared to that in nonsmokers, although there were statistically significant associations only in current smokers among males.

**Table 2:** Results of factors associated between smoking and nonalcoholic fatty liver disease.

**Variables**		**Nonalcoholic fatty liver disease (NAFLD)**			
		**Male**		**Female**	
		**OR**	**95% CI**	**OR**	**95% CI**
**Smoking Behavior**					
	Nonsmoker	1.00		1.00	
	Ex-smoker	1.12	(0.90 – 1.41)	1.32	(0.86 – 2.01)
	Current smoker	1.38	(1.08 – 1.76)	1.18	(0.76 – 1.83)
**Age** **Marital status**		1.00	(1.00 – 1.01)	1.01	(1.00 – 1.83)
	Married	1.00		1.00	
	Divorced, Separated	1.29	(0.80 – 2.07)	1.31	(0.93 – 1.84)
	Single, widow	0.84	(0.64 – 1.10)	0.91	(0.72 – 1.13)
**Educational level**					
	Middle school or below	1.00		1.00	
	High school	1.07	(0.79 – 1.44)	1.03	(0.78 – 1.36)
	College or over	1.06	(0.77 – 1.45)	0.73	(0.53 – 1.01)
**Household income**					
	Low	1.04	(0.74 – 1.44)	0.83	(0.60 – 1.16)
	Mid-low	1.17	(0.92 – 1.49)	0.73	(0.55 – 0.95)
	Mid-high	0.97	(0.78 – 1.21)	0.68	(0.51 – 0.89)
	High	1.00		1.00	
**Region**					
	Metropolitan	1.00		1.00	
	Urban	1.06	(0.87 – 1.30)	1.12	(0.91 – 1.38)
	Rural	1.08	(0.85 – 1.38)	1.34	(1.01 – 1.77)
**Occupational categories**					
	White	0.88	(0.65 – 1.19)	0.81	(0.61 – 1.07)
	Pink	0.78	(0.55 – 1.09)	0.94	(0.72 – 1.23)
	Blue	0.71	(0.55 – 0.92)	0.63	(0.48 – 0.82)
	Inoccupation	1.00		1.00	
**Current drinking status**					
	Never or occasionally	1.00		1.00	
	2–4 times/month	0.89	(0.72 – 1.09)	0.85	(0.70 – 1.04)
	2–4 times/week	1.06	(0.86 – 1.31)	0.60	(0.43 – 0.84)
**Physical activity**					
	Adequate	1.00		1.00	
	Inadequate	1.44	(1.20 – 1.71)	1.33	(1.11 – 1.59)
**BMI**					
	Normal	1.00		1.00	
	Underweight	0.50	(0.14 – 1.78)	0.44	(0.17 – 1.15)
	Overweight	2.84	(2.09 – 3.86)	4.21	(3.28 – 5.41)
	Obesity of stage 1	10.44	(7.94 – 13.72)	11.48	(8.92 – 14.78)
	Obesity of stages 2&3	50.57	(32.58 – 78.48)	62.42	(41.29 – 94.35)
	No	1.00		1.00	
	Yes	1.42	(1.14 – 1.77)	1.96	(1.55 – 2.48)
**Diagnosis of diabetes**					
	No	1.00		1.00	
	Yes	4.36	(3.28 – 5.80)	6.06	(4.41 – 8.31)
**Year**					
	2019	1.00		1.00	
	2020	1.06	(0.88 – 1.27)	0.95	(0.79 – 1.15)

**Figure 2:**
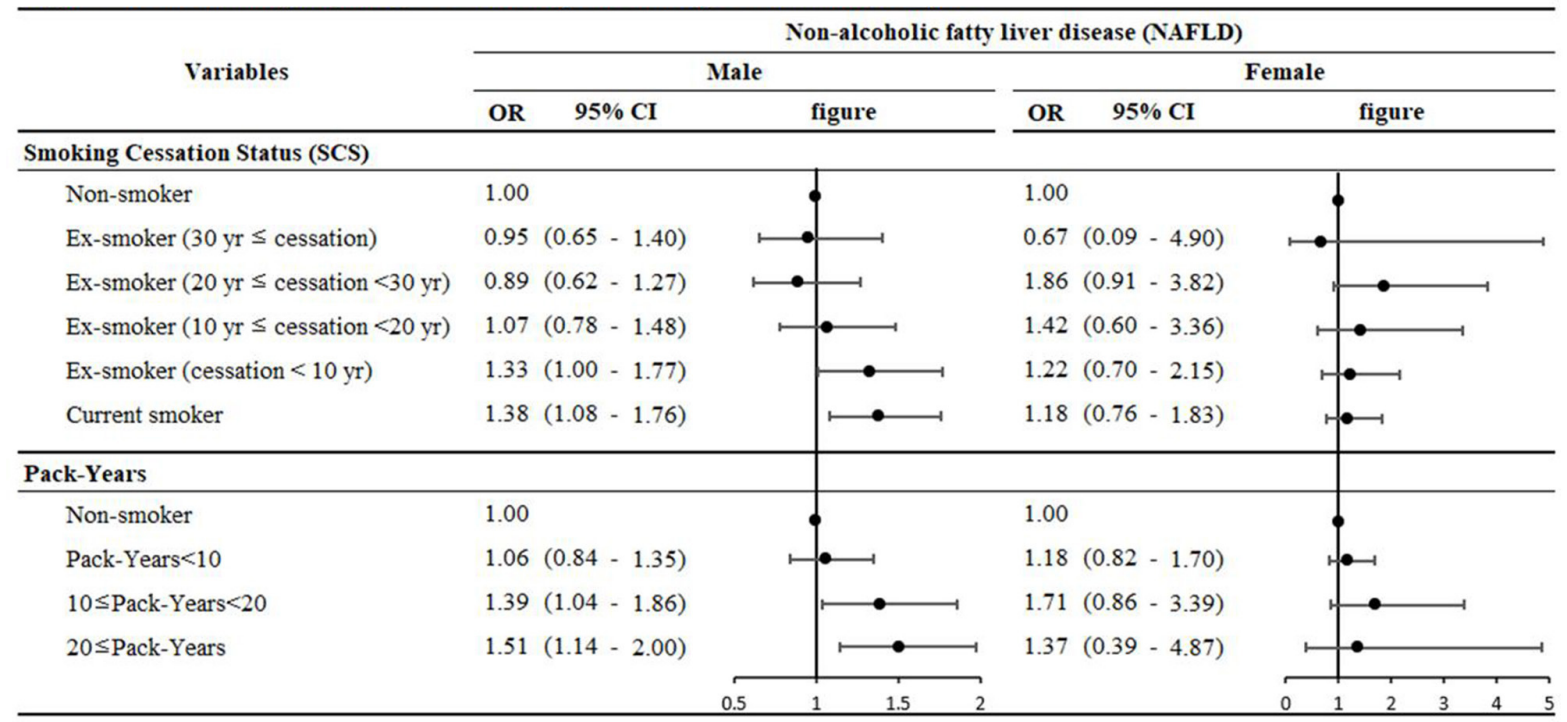
Results of the subgroup analysis stratified by smoking cessation and pack-years.

Figure 2 presents the results of the stratified subgroup analysis of the association between SCS and pack years, indicating the effect of the number of cigarettes and the smoking period on NAFLD according to smoking behavior. In general, with nonsmokers as the reference category, the OR for NAFLD increased linearly as smoking cessation decreased and pack years increased in males. Specifically, an ex-smoker with smoking cessation for < 10 years (OR: 1.33, 95% CI: 1.00–1.77) and a current smoker (OR: 1.38, 95% CI: 1.08–1.76) had the strongest statistically significant association compared to a nonsmoker, as classified based on the smoking cessation period. Furthermore, an ex-smoker and current smoker with 10 to 20 pack years (OR: 1.39, 95% CI: 1.04–1.86) and over 20 pack years (OR: 1.51, 95% CI: 1.14–2.00), respectively, was more likely to have a strong relationship with NAFLD compared to a nonsmoker.

Table 3 shows the results of the independent variable subgroup analysis, representing the ORs for NAFLD stratified by the smoking status. Among current male smokers, cases of never or occasional drinking (OR: 1.78, 95% CI: 1.14–2.78), adequate physical activity (OR: 1.55, 95% CI: 1.09–2.21), BMI indicating overweight (OR: 2.31, 95% CI: 1.40–3.83), no diagnosis of hypertension (OR: 1.42, 95% CI: 1.07–1.87), and no diagnosis of diabetes (OR: 1.39, 95% CI: 1.08–1.79) showed the strongest associations with NAFLD compared to male nonsmokers. In women, drinking 2 to 4 times per month (current smokers: OR: 1.39, 95% CI: 1.08–1.79), normal BMI (ex-smokers: OR: 2.74, 95% CI: 1.28–5.88), and BMI indicating stage 2 and 3 obesity (ex-smokers: OR: 4.36, 95% CI: 1.14–16.71) showed the strongest associations with NAFLD compared to those in nonsmokers.

**Table 3:** Results of subgroup analysis stratified by independent variables.

		**Nonalcoholic fatty liver disease (NAFLD)**										
		**Male**						**Female**				
		**Non**	**Ex-smoker**		**Current smoker**			**Non**	**Ex-smoker**		**Current smoker**	
		**OR**	**OR**	**95% CI**	**OR**	**95% CI**		**OR**	**OR**	**95% CI**	**OR**	**95% CI**
**Age**												
	20–29	1.00	0.53	(0.21 – 1.37)	1.06	(0.54 – 2.09)		1.00	0.82	(0.18 – 3.63)	1.12	(0.32 – 3.92)
	30–39	1.00	0.71	(0.33 – 1.51)	1.18	(0.65 – 2.16)		1.00	1.68	(0.41 – 6.84)	0.62	(0.15 – 2.51)
	40–49	1.00	1.35	(0.71 – 2.58)	1.47	(0.76 – 2.86)		1.00	1.40	(0.61 – 3.22)	0.74	(0.26 – 2.13)
	50–59	1.00	1.14	(0.62 – 2.08)	1.38	(0.72 – 2.62)		1.00	2.67	(1.06 – 6.73)	3.16	(1.12 – 8.86)
	60–69	1.00	1.58	(0.94 – 2.67)	1.51	(0.83 – 2.76)		1.00	0.78	(0.33 – 1.86)	1.07	(0.44 – 2.63)
	≥70	1.00	0.98	(0.58 – 1.66)	0.99	(0.43 – 2.27)		1.00	0.71	(0.18 – 2.85)	0.38	(0.10 – 1.39)
**Current drinking status**												
	Never or occasionally	1.00	0.97	(0.64 – 1.46)	1.78	(1.14 – 2.78)		1.00	1.12	(0.60 – 2.06)	0.61	(0.33 – 1.13)
	2–4 times/month	1.00	1.05	(0.74 – 1.50)	1.21	(0.84 – 1.76)		1.00	1.30	(0.59 – 2.87)	2.28	(1.14 – 4.56)
	2–4 times/week	1.00	1.52	(0.95 – 2.43)	1.56	(1.01 – 2.42)		1.00	2.56	(0.95 – 6.95)	0.91	(0.36 – 2.31)
**Physical activity**												
	Adequate	1.00	1.07	(0.77 – 1.50)	1.55	(1.09 – 2.21)		1.00	1.28	(0.67 – 2.44)	0.99	(0.48 – 2.03)
	Inadequate	1.00	1.15	(0.84 – 1.58)	1.28	(0.90 – 1.83)		1.00	1.33	(0.78 – 2.28)	1.27	(0.74 – 2.19)
**BMI**												
	Underweight	1.00	–	–	–	–	–	1.00	–	–	–	–
	Normal	1.00	1.32	(0.74 – 2.34)	1.14	(0.57 – 2.25)		1.00	2.74	(1.28 – 5.88)	2.16	(0.96 – 4.85)
	Overweight	1.00	1.63	(0.96 – 2.75)	2.31	(1.40 – 3.83)		1.00	0.79	(0.25 – 2.49)	1.04	(0.41 – 2.65)
	Obesity of stage 1	1.00	0.97	(0.71 – 1.33)	1.14	(0.82 – 1.60)		1.00	0.77	(0.38 – 1.57)	1.01	(0.52 – 1.96)
	Obesity of stages 2&3	1.00	0.86	(0.28 – 2.60)	1.73	(0.63 – 4.77)		1.00	4.36	(1.14 – 16.71)	1.21	(0.25 – 5.81)
**Diagnosis of hypertension**												
	No	1.00	1.07	(0.82 – 1.41)	1.42	(1.07 – 1.87)		1.00	1.33	(0.81 – 2.19)	1.28	(0.79 – 2.07)
	Yes	1.00	1.16	(0.74 – 1.82)	1.05	(0.62 – 1.78)		1.00	1.35	(0.61 – 2.99)	0.90	(0.34 – 2.37)
**Diagnosis of diabetes**												
	No	1.00	1.06	(0.83 – 1.35)	1.39	(1.08 – 1.79)		1.00	1.29	(0.83 – 2.01)	1.22	(0.76 – 1.95)
	Yes	1.00	1.79	(0.85 – 3.80)	0.91	(0.39 – 2.13)		1.00	2.14	(0.24 – 19.16)	1.19	(0.30 – 4.67)

## Discussion

The general findings were that there is an association between smoking and NAFLD, and the risk of having NAFLD has a dose-dependent negative association with the duration of smoking cessation and a positive association with pack years. Given these results, our study suggests that ex-smokers with an SCS of fewer than 10 years had associations similar to those seen in current smokers, while ex-smokers whose SCS was more than 20 years had no association. Furthermore, we found a strong linear association between the duration of smoking and the number of cigarettes smoked per day. These findings are consistent with the results of a previous study (30) and may provide supporting evidence for an association between smoking history and NAFLD. Smoking cessation reduces the incidence of NAFLD. However, due to the low number of female smokers in Korea, we could not find a relationship between smoking and NAFLD among females. However, although not statistically significant, the OR of former smokers and current smokers was higher than that of nonsmokers. This reflects the recall bias of self-reported data due to the poor perception of female smokers in Korea (31).

Smoking has been identified, as an adjunct to obesity, as a causative factor for NAFLD in animal and clinical studies (25, 32). This study found no association between smoking behavior and NAFLD in men with stages 1, 2, and 3 obesity; however, in overweight men and normal women, smoking behavior was a significant risk factor associated with NAFLD compared to nonsmoking. This supports the results of a previous study (33) suggesting that while severe obesity directly affects NAFLD in BMI groups, smoking may have an independent relationship in normal or overweight groups. A mechanism that explains the independent role of BMI in the association between smoking and NAFLD is that the antiestrogenic effect of cigarette smoking leads to a change in body fat distribution (34–36). Therefore, normal and overweight smokers who may not be evaluated for NAFLD should receive more attention to prevent NAFLD.

According to the multiple parallel hits hypothesis theory, the pathophysiological mechanisms of NAFLD indicate the causes of insulin resistance, genetic and epigenetic factors, mitochondrial dysfunction, endoplasmic reticulum stress, microbiota, chromatic low-grade injury, and dysfunction of adipose tissue (37, 38). In insulin-resistant patients, liver fat production can be further induced by activation of transcription factors such as SREBP-1 (38, 39). Many studies have shown that tobacco increases lipid accumulation in liver cells by regulating the activity of 5′-AMP-activated protein kinase (AMPK) and SREBP-1, two important molecules involved in lipid synthesis (27, 40–42). It is considered a mechanism between smoking and NAFLD, especially based on previous studies that show a decisive role in liver fat accumulation in SREBP-1, when tobacco smoke is exposed to mice and cultured hepatocytes (27).

However, the effects of smoking on NAFLD remain controversial, with inconsistent results (43). One study reported that active smoking was associated with fibrosis in patients with NAFLD (25), but another study showed a lack of significant relationship between active smoking and NAFLD (44). Several experimental studies in mice have shown that nicotine, a dangerous substance in cigarettes, promotes the development of NAFLD or accelerates its progression (25–27). A systematic review and meta-analysis of 20 observational studies showed that smoking was significantly associated with NAFLD (43). Furthermore, second-hand smoking increases the risk of NAFLD around 1.38 times (43). Based on these mechanisms, experimental studies and cohort studies that consider additional confounders are needed.

This study had several limitations. First, it was a cross-sectional study. It may not establish temporal relations and may have found an inverse causal relationship. Therefore, caution is warranted when interpreting the results. More research is needed to clarify the association between smoking and NAFLD. Second, KNHANES data were collected through self-report surveys. Hence, data on health-related status, socioeconomic variables, and smoking status may not be reliable and accurate. In particular, this can lead to recall bias and is likely to be underestimated in the case of smoking. Third, although the liver fat score for NAFLD was demonstrated for the ROC curves for detecting NAFLD (sensitivity of 86% and specificity of 71%), there were still tiny errors of false-positive or false-negative results. In addition, due to the characteristics of the KNHANES called secondary data, the diagnosis of NAFLD was not measured by the instrument investigation, so steatosis could not be confirmed by methods such as CAP, ultrasound, and liver biopsy. Therefore, we calculated and considered the NAFLD liver fat score instead. Fourth, it could not differentiate among the various smoking types, such as conventional cigarette use, electronic cigarette use, or both. Besides, we could not calculate the pack years for e-cigarettes because the KNHANES did not include this information. Finally, we cannot exclude the possibility of unrecognized confounders, although we adjusted for known confounders in the relationship between smoking and NAFLD.

Despite these limitations, our study had several notable strengths. First, the study was based on the KNHANES data, a nationally representative dataset collected by the KDCA. This is useful for health-related research because it is updated annually to reflect the changes in the actual health situation of Koreans. In addition, it is a statistic that can generalize the study results to the general population because the survey is performed by reliable and representative random cluster sampling. Second, we calculated the SCS and pack years for ex-smokers and current smokers. The study showed a significant association between current smoking behavior in men and smoking status considering SCS and pack-years. Therefore, our results suggest that smoking status has the opportunity to be considered as a measure of intervention to reduce the risk of NAFLD when SCS and pack years are taken into account.

## Conclusion

In conclusion, this study found that current smoking was associated with NAFLD in men in the South Korean population. In particular, we suggest an association between NAFLD and ex-smoker and current smoker status with a short smoking cessation period or many pack years. Given these results, smoking has a potential effect on NAFLD, and smoking cessation should be considered in the prevention and management of NAFLD. It is best to stop smoking considering health status and behavior to avoid serious diseases. More prospective studies and clinical trials are required to clarify the relationship between smoking history and NAFLD.

## Data availability statement

Publicly available datasets were analyzed in this study. This data can be found here: https://www.kdca.go.kr/index.es?sid=a2.

## Ethics statement

Ethical review and approval was not required for the study on human participants in accordance with the local legislation and institutional requirements. Written informed consent for participation was not required for this study in accordance with the national legislation and the institutional requirements.

## Author contributions

YSJ designed the study, collected data, performed statistical analysis, and drafted the manuscript. YSJ, HJJ, YSP, E-CP, and S-IJ contributed to the discussion. S-IJ is the guarantor of this work, has full access to all the study data, and assumes responsibility for the integrity of the data and the accuracy of the data analysis. All authors reviewed and edited the drafts of the manuscript, approved the final version, and approved the final manuscript.
